# Does the acceptance of hybrid learning affect learning approaches in France?

**DOI:** 10.3352/jeehp.2017.14.24

**Published:** 2017-10-20

**Authors:** Lionel Di Marco, Alain Venot, Pierre Gillois

**Affiliations:** 1 TIMC-IMAG UMR 5525, Themas, CNRS, Grenoble-Alpes University, Grenoble, France; 2 Midwifery Department, Faculty of Medicine, Grenoble-Alpes University, Grenoble, France; 3 LIMICS, UMRS 1142 & Paris 13 University & UPMC Paris 6 University, Paris, France; Hallym University, Korea

**Keywords:** Educational models, Undergraduate medical education, Teaching materials, Learning approach, Learning strategies, Technology acceptance

## Abstract

**Purpose:**

Acceptance of a learning technology affects students’ intention to use that technology, but the influence of the acceptance of a learning technology on learning approaches has not been investigated in the literature. A deep learning approach is important in the field of health, where links must be created between skills, knowledge, and habits. Our hypothesis was that acceptance of a hybrid learning model would affect students’ way of learning.

**Methods:**

We analysed these concepts, and their correlations, in the context of a flipped classroom method using a local learning management system. In a sample of all students within a single year of study in the midwifery program (n= 38), we used 3 validated scales to evaluate these concepts (the Study Process Questionnaire, My Intellectual Work Tools, and the Hybrid E-Learning Acceptance Model: Learner Perceptions).

**Results:**

Our sample had a positive acceptance of the learning model, but a neutral intention to use it. Students reported that they were distractible during distance learning. They presented a better mean score for the deep approach than for the superficial approach (P< 0.001), which is consistent with their declared learning strategies (personal reorganization of information; search and use of examples). There was no correlation between poor acceptance of the learning model and inadequate learning approaches. The strategy of using deep learning techniques was moderately correlated with acceptance of the learning model (r_s_= 0.42, P= 0.03).

**Conclusion:**

Learning approaches were not affected by acceptance of a hybrid learning model, due to the flexibility of the tool. However, we identified problems in the students’ time utilization, which explains their neutral intention to use the system.

## Introduction

**Fig. 1.**
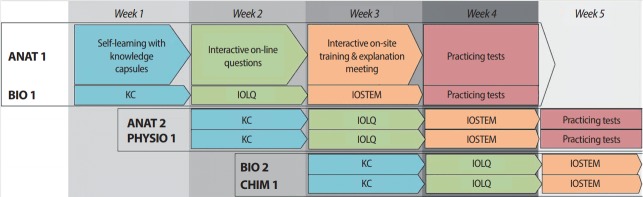
Stepwise learning method in sequences of 4 activities. During each week, students work on 4 themes, each in different ways: KC, IOLQ, IOSTEM, and practice tests. KC, knowledge capsules; IOLQ, interactive online questions; IOSTEM, interactive on-site training and explaining meetings.

Blended learning based on a flipped classroom has many points of interest: students are multi-dimensionally satisfied [1], it improves students’ work conditions and teaching methods, and it facilitates active learning [2]. The effectiveness of these pedagogical methods in the field of health has already been proven [3,4] and the use of these methods as a key tool in the learning paradigm is continually increasing [5]. The University of Grenoble decided in 2006 to use a flipped classroom model during the first year of health studies, in a program named “PACES.” This innovative pedagogical method is also used for the second year of study (since 2007 for midwifery students, and since 2011 for medical and pharmacy students). A 4-step learning method was proposed for students to learn (Fig. 1), in which each theme is split to fit the 4 sequential weeks of learning: remotely-accessible knowledge capsules, interactive online questions for the lecturer, interactive on-site training and explanation meetings with the lecturer, and participation in a training-exam session or practice tests with junior tutors (graduate students).

The method is supported by a local learning management system (LMS), and utilizes the classic features of an LMS: access to different learning activities (remotely-accessible lectures, self-assessment, online questions, SCORM-format activities, collaborative techniques, etc.), and a planner for learning activities that schedules and provides access to learning activities at specific times.

Since 2006, between 1,600 and 2,000 students have annually registered in the first year of health studies (PACES). Including the paramedic students and the other years of the medical departments, in 2016, this method reached 10,000 students. This learning model is used in most of the areas of health studies at Grenoble-Alpes University, but with different objectives according to the year of the curriculum. Some students must succeed on a very competitive exam for which they are trained each week during 1 or more years (in PACES), and some other students, as in midwifery, must learn a health profession over the course of years, and are required to make links between knowledge, skills, and habits. With this objective of making links, a deep learning approach (with understanding and active learning of information) seems to be the most promising learning approach for health professions students. According to Biggs et al. [6], the choice of the learning style is influenced by the learning context in which students are evolving.

For more than 10 years, Grenoble-Alpes University has used blended learning in most fields in health and all years of the curriculum. However, the way students learn is unknown, as is the way that students perceive the learning model. The main objective of our study was to describe the learning process of students engaged in the health curriculum, in order to identify their perceptions and their strategies that they use in the context of this specific pedagogical model. The secondary objective was to determine whether any correlation existed between a poor acceptance of the model and inadequate learning approaches, as a way of evaluating this educational method.

## Methods

### Population

Differences in learning objectives exist between year 1 (PACES) and year 2 of Grenoble’s medical school. The population with the longest experience of this method is midwifery students. We carried out an observational, prospective, monocentric study based on student-centred evaluations. Our survey was composed of 4 main parts to measure the 3 main concepts and to evaluate how midwifery students used the LMS during year 2. Our primary outcomes were the mean results of the scores described below. Our secondary outcomes were the existing correlations between the concepts that were analysed.

### Concept

***Acceptance of a hybrid e-learning model:*** Ahmed suggested that acceptance of hybrid e-learning courses is related to information technology infrastructure, support, and instructor characteristics [7]. He based his work on Davis’ Technology Acceptance Model (TAM, 1989), and created the Hybrid E-Learning Acceptance Model: Learner Perceptions (HELAM-L). The validity and reliability of this model have been established. We chose this model because the TAM has been already used with the midwifery students in Grenoble, in the context of a local study. Moreover, our study was an opportunity to test the HELAM-L in a context different from that in which it was created.

***Learning approaches:*** Learning approaches were first studied by Biggs, who developed a scale for evaluating students’ learning approaches: the Study Process Questionnaire (R-SPQ-2F). This questionnaire analyses students’ motivations and strategies to determine their learning approach (superficial or deep). We used the French translation of this questionnaire, which has been validated by several authors [8].

***Learning strategies:*** To complete our analysis of learning approaches, we decided to define students’ learning strategies clearly. Wolfs designed and validated a scale named “My Intellectual Work Tools” (our translation from French) that evaluates declared cognitive behaviours, used metacognitive skills, emotional factors, and motivations [9]. The authors identified 10 learning techniques used by students (detailed in Table 1).

### Statistics

Statistical analysis was performed using R ver. 3.4 (https://www.r-project.org/). All data were recorded in an online LimeSurvey database. The descriptive statistics primarily present percentages per class and 95% confidence intervals (CIs) or median and interquartile range (IQR). As an analytic tool, the Spearman coefficient (r_s_) was used to analyse the correlations between acceptance of the pedagogical model and learning approaches and strategies. When studying psychological concepts, r_s_= 0 shows no correlation, 0< r_s_< 0.4 shows a weak correlation, 0.4≤ r_s_< 0.7 shows a moderate correlation, 0.7≤ r_s_< 1 shows a strong correlation, and r_s_= 1 shows a perfect correlation. We used a significance level of the Spearman correlation test where α=0.05.

### Ethical approval

Informed consent was provided by the subjects. An institutional review board (IRB) exemption was approved for this study (No. IRB-00010290) by the IRB of COMUE Université Grenoble Alpes because it did not include any individual identification.

## Results

### Population

The population consisted of the full class of year 2 of the midwifery curriculum (38 students). The raw data are available in Supplement 1. The participation rate was 68.4% (n= 26). The median age was 20 years (IQR, 0.75). Sixty-seven percent chose midwifery as their first choice of a career. To the question “How do you estimate your computer skills? Very good/good/deficient/poor”, the median response was “good.”

**Fig. 2.**
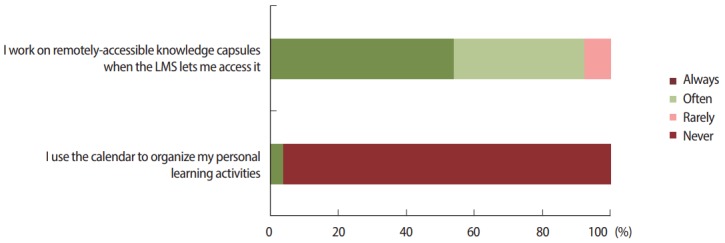
Most students never used the planner to organize their work, but often or always followed the organization proposed by the LMS. They used the default tool (a raw list of all knowledge capsules) instead of the organizational tool (a precise planner of all the learning activities). LMS, learning management system.

**Fig. 3.**
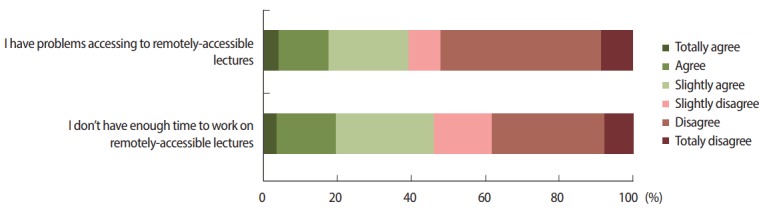
Forty percent of students had problems accessing knowledge capsules and 50% did not have enough time to work on them.

Concerning their use of the LMS, such as managing their learning activities, most of them never used the planner to organize their work (Fig. 2); 46.1% (95% CI, 27–65.3) disagreed with the statement that they had enough time to work on remotely-accessible knowledge capsules before classroom sessions (declaring themselves to be easily distracted during this activity), and 38.4% (95% CI, 42.8–80.2) agreed with the statement that they had problems accessing the remotely-accessible knowledge capsules (Fig. 3).

### Acceptance model

**Fig. 4.**
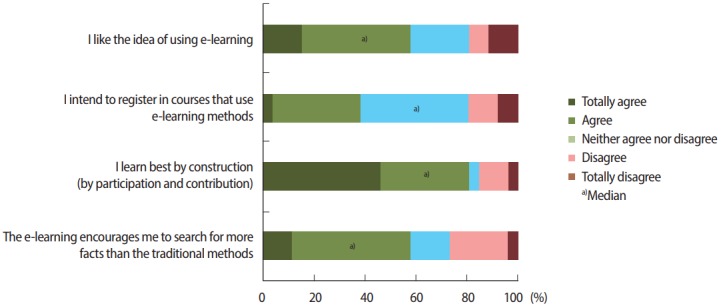
Students had a slightly positive acceptance of the pedagogical model, but their intentions to use e-learning methods were neutral. The Likert scale used in the Hybrid E-Learning Acceptance Model contains the following items: totally agree, agree, neither agree nor disagree, disagree, and totally disagree.

According to the HELAM-L, we found that our population had a slightly positive acceptance of this pedagogical model (Table 2). The midwifery students found the instructor to be encouraging, effective, enthusiastic, and clarifying. They found the infrastructure to be accessible, but support did not seem to be easily accessed (i.e., there was a lack of computer stations and printers). Our population had a slightly positive acceptance of the model, but students’ intentions to use the method were neutral (Fig. 4).

### Learning approaches and learning strategies

Concerning students’ learning approaches, the mean scores on the R-SPQ-2F survey showed that our population had higher scores for the deep approach than for the superficial approach (maximum score, 25) concerning strategy (P< 0.05), motivations (P< 0.001), or both (P< 0.001). Concerning their learning strategies, memorization by heart was the least used technique, while the personal reorganization of information was the most used (Table 1).

### Links between acceptance and learning approaches

**Fig. 5.**
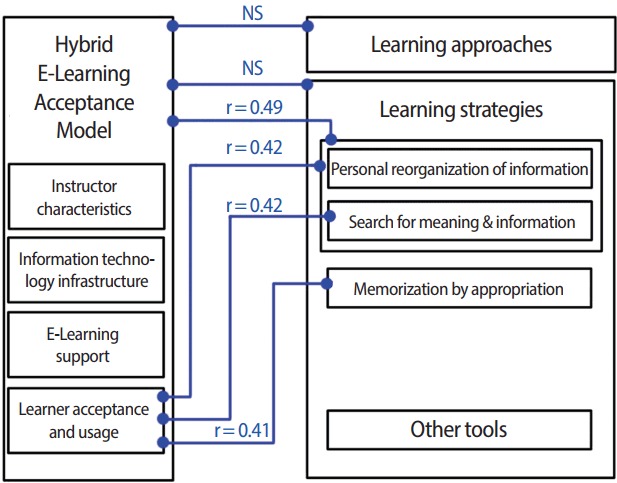
Declared use of deep learning techniques were correlated with learners’ acceptance of the hybrid model, but learning approaches were not signifcantly correlated with acceptance. The most interesting correlations, signifcant or not, are presented in this fgure. NS, not signifcant.

There was no significant correlation between acceptance and learning approaches (r_s_<0.2, P>0.05; not significant [NS]). Stronger correlations were found with an acceptance below the average score, but this was not significant (r_s_= 0.42, P> 0.05; NS) (Fig. 5).

We found a moderately significant correlation between acceptance of the model and the strategy of searching for meaning (r_s_= 0.42, P= 0.03; significant [S]). We also found a moderately significant correlation between acceptance and active learning (r_s_= 0.41, P= 0.03; S). The other correlations were not significant.

Using deep learning technique (technique 1+technique 5) was also moderately but significantly correlated with the acceptance of the model (r_s_= 0.42, P= 0.03; S) and to the global HELAM-L score (r_s_= 0.49, P= 0.01; S).

## Discussion

### Positive acceptance of the pedagogical model and students’ deep learning

Students declared that they were easily distracted (by the telephone, naps, social networks, TV series, etc.), and griped about the lack of computer workstations and printers. To prevent this distractibility, all personal learning activities are outlined in the planner, with online access to the resources. That means that for these activities, as for face-to-face activities, a specific time is determined and indicated in the online planner. It may be that students need a specific environment to engage in their personal learning activities on site or at school during a dedicated time. They had a slightly positive acceptance of this pedagogical model, but their intentions to use this learning method were neutral.

Unfortunately, the midwifery students did not use the LMS as intended. During midwifery studies, not all the lectures are “flipped,” and time management is therefore very important to manage both types of lectures. Students’ organizational skills can clearly be improved, because they stated that they did not have enough time to learn the remotely-accessible knowledge capsules. They did not use the planner as intended, and instead only used the default course syllabi (without any time specification). This global misuse did not interfere with the acceptance of the method or with their perceptions of the information communication technology (ICT) tool.

The students understood that they must comprehend and actively learn knowledge and basic skills; in other words, they recognized that they were learning a job, not competing in a contest. The widespread use of deep learning techniques (such as searching for meaning and personal reorganization of information) explains the non-existent correlation between poor acceptance of the model and inadequate learning approaches. This type of learning approach can also improve their results on exams, as the joint effect of the blended learning activities is positively linked to the students’ final marks [10].

However, a moderate correlation was found between positive acceptance of the model and the use of deep learning techniques. This means that students who tried to understand and actively learn knowledge, compared with those who learned by heart, had a better acceptance of this pedagogical model. This was also true for students who used the technique of searching for meaning. This is a logical result, as students who use the LMS prefer to decide when and how they carry out their personal learning activities. Moreover, they probably develop their personal organizational and time management skills concerning remote coursework courses after 1 or 2 years of PACES, during which they used this pedagogical model.

### A flexible pedagogical model that can be improved

Half of our student sample declared that they had difficulties in assessing themselves. They rarely learned by asking themselves questions, and they reported having difficulties with arguing for their point of view. Our syllabi could be improved, as only a few knowledge capsules incorporate online self-assessment surveys as part of the learning sequence. Even if it seems that self-assessment has yet to have a proven effect on students’ performance [11], it does help students to analyse their own work [12]. Therefore, it seems necessary to develop self-assessment as a personal e-learning activity.

### A specific learning situation and specific benefits

Our results are consistent with those of other studies concerning the benefits of deep learning for similar samples, as shown by Mann’s systematic review [13]. Deep learning seems the most fruitful approach for health education, given the specificity of health professional educational programs [14]. The main limitation of our study was the relatively high participation rate and the small size of the sample (due to the size of the second-year midwifery class). Validated scales were used to analyse the concepts of this study, leading to logical and coherent results.

Our analysis concerns a learning situation that is not generally reproducible. However, blended learning is becoming more and more developed, and our study showed that learning strategies were linked to usage of an LMS and intentions to use a LMS. Evaluating an educational method by analysing and improving an LMS can have positive impacts on students’ learning.

Our results most likely reflect our students’ motivations to learn, as the students predominantly chose midwifery as their first choice of a career. Thus, they are probably interested in understanding how theoretical concepts can be linked to their hands-on experience. In their responses to Wolfs’ scale [9], the midwifery students showed that they used active learning while working on knowledge capsules (highlighting the key content; connecting parts of a text, understanding before memorizing, imagining in what context studied concepts can be useful, etc.). However, they also declared that they often tried to memorize the entire lesson. This is most likely linked to the PACES program and the competitive exam, which requires memorization of the details of the knowledge capsules for students to be able to answer the maximum number of multiple-choice questions correctly [15].

**Fig. 6.**
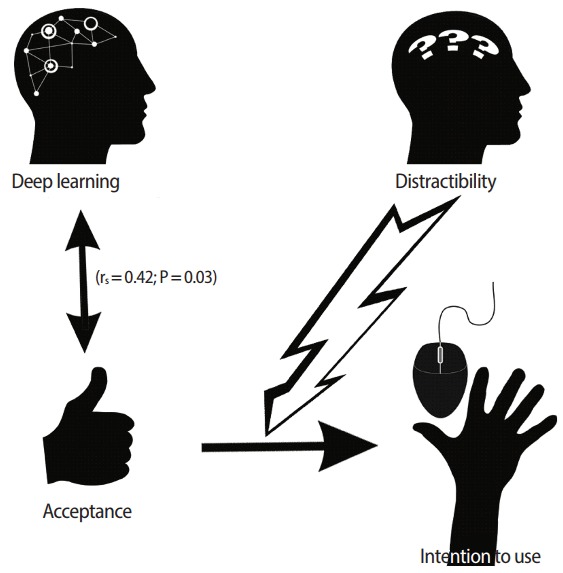
Intention to use was moderately linked to deep learning, but was probably perturbed by distractibility. r_s_, Spearman coefficient.

Our study found that midwifery students learned with a deep approach and deep learning techniques, according to the institutional goals and methods. The blended learning method was accepted by students who declared that they were distracted and had difficulties managing their schedule, even if they did not use the LMS as intended and even if their intentions to use it were neutral (Fig. 6). This hybrid method, including a flipped classroom and a specific LMS, associated with a real choice to learn midwifery, is a motivation for knowledge enhancement. This learning environment is flexible enough to allow students to organize their personal work from fully guided to autonomous learning.

Their intention of use was too neutral for a tool that they are encouraged to use. It would be interesting to improve it with more self-assessment tests. However, if students are required to perform more self-assessment tests, their difficulties in time management may increase. A fine-grained analysis of learning outcomes or of the reasons for students’ distractibility would help identify ways to improve this innovative, efficient, and flexible pedagogical method.

## Figures and Tables

**Table 1.** Deep learning techniques were the most commonly used

Variable	Proportion (%)	95% Confidence interval
Personal reorganization of information (tool 5)	94.2	83.6-102.9
Search for examples and use of examples (tool 3)	90.4	79.0-101.7
Search for meaning and search for information (tool 1)	80.0	64.6-95.4
Active learning (tool 6)	79.5	64.0-95.0
Reformulation (tool 2)	78.2	62.3-94.1
Highlighting of relationships and structure of the course (tool 4)	73.1	56.0-90.1
Time management (tool 8)	68.3	50.4-86.2
Self-assessment and self-confidence (tool 9)	59.0	40.1-77.9
Anticipation of evaluation conditions (tool 7)	56.7	37.7-75.8
Involvement in studies (tool 10)	51.6	32.4-70.9
No. of difficulties (tool 9)	35.4	17.0-53.8
Memorization by heart (tool 6)	26.9	9.9-44.0

These learning techniques are described by Wolfs’ scale “My intellectual work tools” [9].

**Table 2.** Students had a slightly positive acceptance of the pedagogical model

Statement categories	Median
Instructor characteristics	Agree
Information technology infrastructure	Agree
E-learning support	Neither agree nor disagree
Learner acceptance and usage	Neither agree nor disagree & agree

The Likert scale used in the Hybrid E-Learning Acceptance Model contains the following items: totally agree, agree, neither agree nor disagree, disagree, and totally disagree.

## Supplementary materials

Supplement 1. Data file is available from https://doi.org/10.7910/DVN/U4NLCM.

Supplement 2. Audio recording of the abstract.
